# Supplementary material for: Conditional Loss of the Exocyst Component Exoc5 in Retinal Pigment Epithelium (RPE) Results in RPE Dysfunction, Photoreceptor Cell Degeneration, and Decreased Visual Function
Source: Int J Mol Sci. 2021 May 11;22(10):5083. doi: 10.3390/ijms22105083 (PMC8151988; doi:10.3390/ijms22105083)
Supplement: Supplementary file 1 [file ijms-22-05083-s001.zip › ijms-1192444-supplementary.pdf]

SUPPLEMENTARY FIGURES and LEGENDS

Conditional loss of the exocyst component Exoc5 in retinal pigment epithelium (RPE) results in RPE dysfunction, photoreceptor cell degeneration, and decreased visual function.

Bärbel Rohrer[1,2#], Manas R. Biswal[3], Elisabeth Obert[1], Yujing Dang[4], Yanhui Su[4], Xiaofeng Zuo[4], Ben Fogelgren[5], Altaf A. Kondkar[6], Glenn P. Lobo[1,4,7*#] and Joshua H. Lipschutz[1,8#]

[1]Department of Ophthalmology, Medical University of South Carolina, Charleston, SC, 29425, USA.
[2]Ralph H. Johnson VA Medical Center, Division of Research, Charleston, SC 29401. USA.
[3]Department of Pharmaceutical Sciences, Taneja College of Pharmacy, University of South Florida, Tampa, FL 33612, USA.
[4]Department of Medicine, Medical University of South Carolina, Charleston, SC, 29425, USA.
[5]Department of Anatomy, Biochemistry, and Physiology, University of Hawaii at Manoa, Honolulu, HI 96813, USA.
[6]Department of Ophthalmology, College of Medicine, King Saud University, Riyadh 11411, Saudi Arabia.
[7]Department of Ophthalmology and Visual Neurosciences. Lions Research Building, 2001 6th Street SE., Room 225, University of Minnesota, Minneapolis, MN 55455, USA.
[8]Department of Medicine, Ralph H. Johnson Veterans Affairs Medical Center, Charleston SC, 29425, USA.

***Corresponding Author**
Glenn P. Lobo, Ph.D.
Associate Professor
Department of Ophthalmology and Visual Neurosciences
Lions Research Building, 2001 6th Street SE.
Room LRB 225
University of Minnesota, Minneapolis, MN 55455, USA.
E-mail: lobo0023@umn.edu.

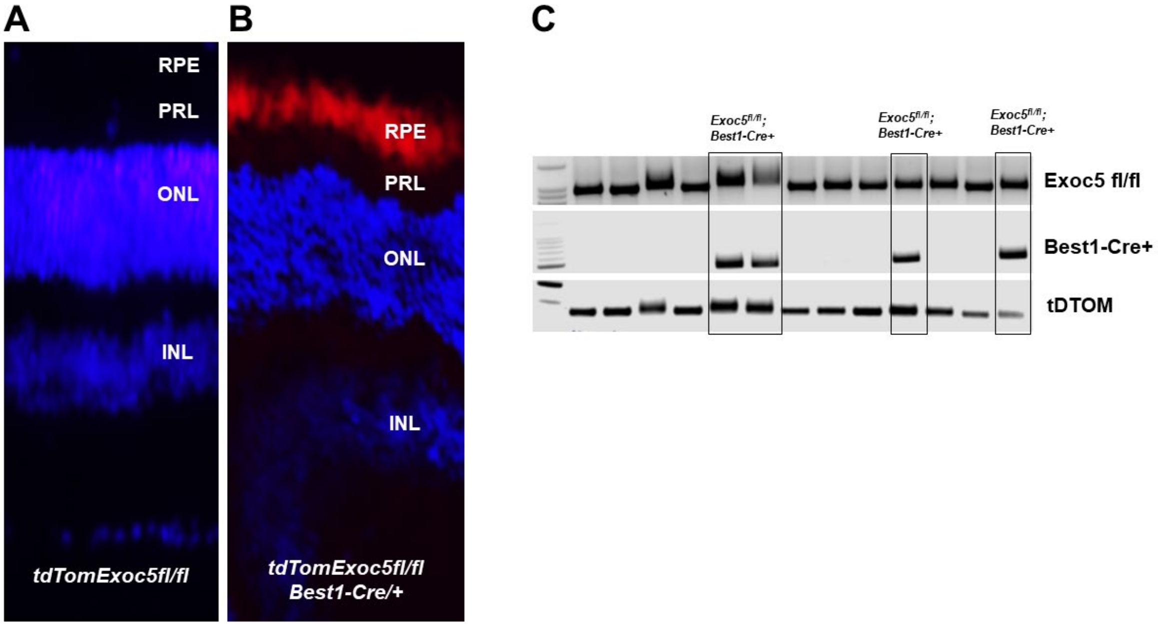

Supplementary Fig. S1: Generation of *tdTomatoExoc5fl/fl* mice and genotyping for conditional *tdTomatoExoc5fl/fl;Best1-Cre*$^{+}$ mice. (**A**) No tdTomato expression (red) was seen in *tdTomatoExoc5fl/fl* mice, (**B**) while tdTomato expression (red color in RPE) occurred in *tdTomato Exoc5fl/fl;Best1-Cre/+* mice, confirming conditional loss of EXOC5 in RPE cells. (**C**) PCR based genotyping for identification of control *tdTomatoExoc5fl/fl* and experimental *tdTomatoExoc5fl/fl;Best1-Cre/+* mice.

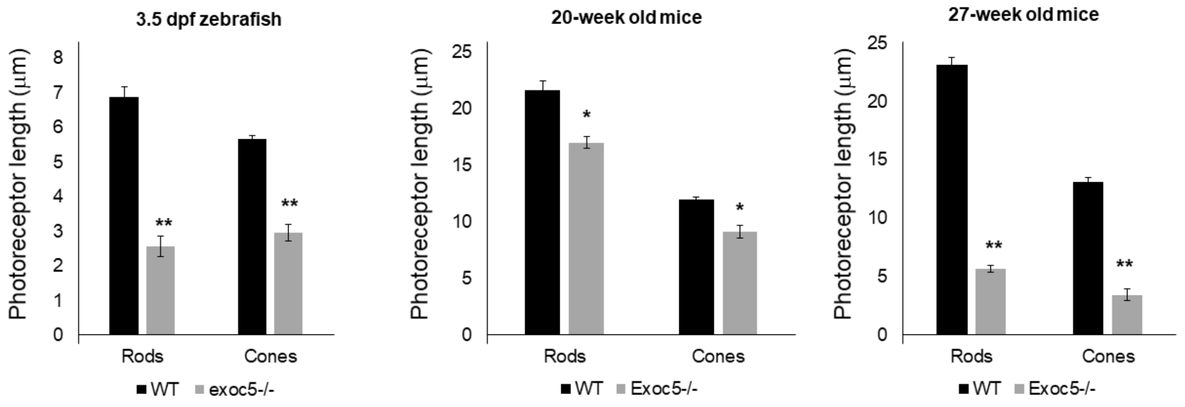

Supplementary Fig. S2: Quantification of photoreceptor lengths. (**A**) 3.5 days post fertilization (dpf) zebrafish larvae, (**B**) 20-weeks old *Exoc5*$^{-/-}$ and WT mice, and (**C**) 27-weeks old *Exoc5*$^{-/-}$ and WT mice.

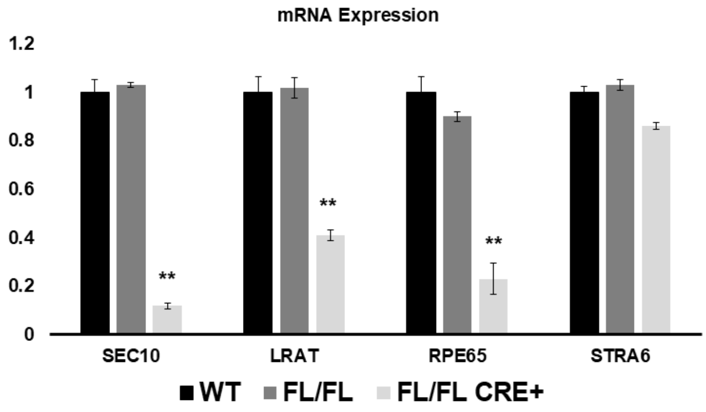

Supplementary Fig. S3: mRNA quantification of key retinal genes involved in vitamin A transport and metabolism. Retinas (n=4) from 27-week old *Exoc5*$^{-/-}$ (Fl/Fl Cre+ experimental) and WT and Exoc5 Fl/Fl (control) mice, were pooled and analyzed by real-time qRTPCR. ** $p<0.005$.

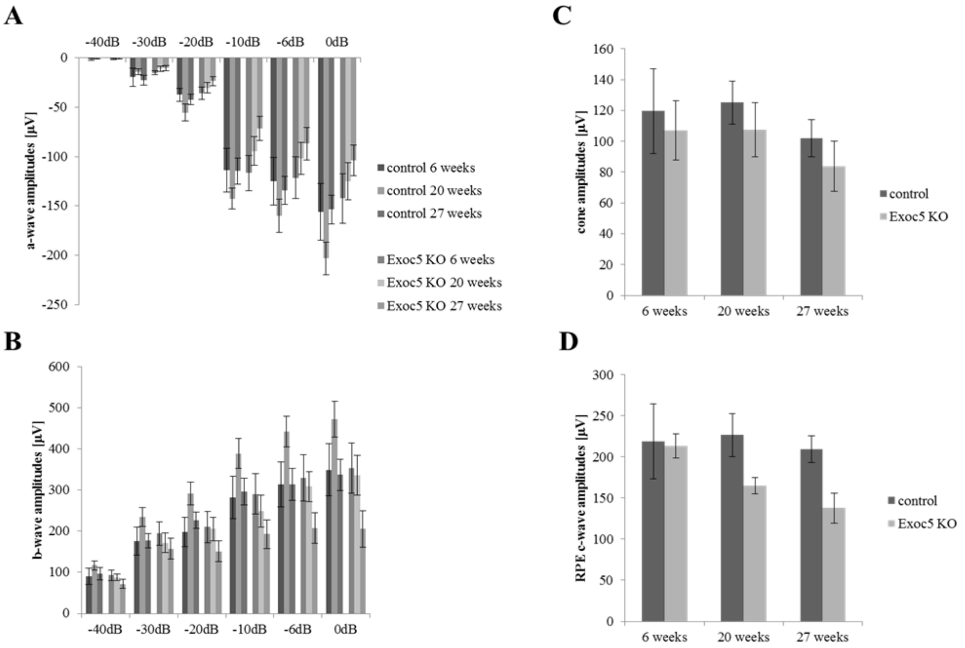

Supplementary Fig. S4: Measurement of visual function in *Exoc5fl/fl;Best1-Cre*⁺ mice by full-field Electroretinography (ERG) and *c*-wave function. Data are expressed as mean ± SEM (*Exoc5 fl/fl;Best1 -Cre*⁻: n=10; and *Exoc5 fl/fl;Best1 -Cre*⁺ mice: n=10).
